# Standing variation and new mutations both contribute to a fast response to selection for flowering time in maize inbreds

**DOI:** 10.1186/1471-2148-10-2

**Published:** 2010-01-04

**Authors:** Eléonore Durand, Maud I Tenaillon, Céline Ridel, Denis Coubriche, Philippe Jamin, Sophie Jouanne, Adrienne Ressayre, Alain Charcosset, Christine Dillmann

**Affiliations:** 1 INRA, UMR de Génétique Végétale, INRA/CNRS/Univ Paris-Sud/AgroParistech, Ferme du Moulon, F-91190 Gif sur Yvette, France; 2 CNRS, UMR de Génétique Végétale, INRA/CNRS/Univ Paris-Sud/AgroParistech, Ferme du Moulon, F-91190 Gif sur Yvette, France; 3 Univ Paris-Sud, UMR de Génétique Végétale, INRA/CNRS/Univ Paris-Sud/AgroParistech, Ferme du Moulon, F-91190 Gif sur Yvette, France

## Abstract

**Background:**

In order to investigate the rate and limits of the response to selection from highly inbred genetic material and evaluate the respective contribution of standing variation and new mutations, we conducted a divergent selection experiment from maize inbred lines in open-field conditions during 7 years. Two maize commercial seed lots considered as inbred lines, *F*252 and *MBS*847, constituted two biological replicates of the experiment. In each replicate, we derived an Early and a Late population by selecting and selfing the earliest and the latest individuals, respectively, to produce the next generation.

**Results:**

All populations, except the Early *MBS*847, responded to selection despite a short number of generations and a small effective population size. Part of the response can be attributed to standing genetic variation in the initial seed lot. Indeed, we identified one polymorphism initially segregating in the *F*252 seed lot at a candidate locus for flowering time, which explained 35% of the trait variation within the Late *F*252 population. However, the model that best explained our data takes into account both residual polymorphism in the initial seed lots and a constant input of heritable genetic variation by new (epi)mutations. Under this model, values of mutational heritability range from 0.013 to 0.025, and stand as an upper bound compare to what is reported in other species.

**Conclusions:**

Our study reports a long-term divergent selection experiment for a complex trait, flowering time, conducted on maize in open-field conditions. Starting from a highly inbred material, we created within a few generations populations that strikingly differ from the initial seed lot for flowering time while preserving most of the phenotypic characteristics of the initial inbred. Such material is unique for studying the dynamics of the response to selection and its determinants. In addition to the fixation of a standing beneficial mutation associated with a large phenotypic effect, a constant input of genetic variance by new mutations has likely contributed to the response. We discuss our results in the context of the evolution and mutational dynamics of populations characterized by a small effective population size.

## Background

Quantifying the proportion of genetic variability that can be attributed to new mutations is a central question in evolutionary quantitative genetics [1-3]. Mutational genetic variance defines the range of variation that can be explored by a population facing new environmental conditions and ultimately determines the rate of evolution of a population [4]. This mutational genetic variance both depends on the mutation rate and on the phenotypic consequences of the mutations (the mutational effects). In particular, theoretical models predict that the amount of total expected genetic variance for a trait at selection/mutation/drift equilibrium is heavily dependent on the shape of the distribution of mutational effects [5-8]. Numerous empirical studies have been undertaken to measure the rate and distribution of mutational effects in a variety of model organisms. These studies are either based on comparative analysis of sequences from different species, or on Mutation and Selection experiments.

Comparative analyses of sequences rely on the comparison of species pairs with known divergence and aim at identifying loci under selection during the past history of a species and more generally emphasized the role of selection in shaping molecular polymorphism patterns. The approach uses derivatives of the Mc Donald-Kreitman test [9] based on the comparison between the variation within species (polymorphism) and the divergence at both synonymous and non synonymous sites. An interesting outcome of this approach is the variation between the estimates of the rate of adaptive substitutions in different species. Actually, the proportion of loci that have been submitted to adaptive evolution ranges from a few percent in *Arapidopsis thaliana *and Human, up to ≈ 50% in *Drosophila *and microorganisms (for a review, see [10]). Comparative analyses of sequences have also been used to estimate the genomic rate of deleterious mutation *U*. From a comparison of 46 protein-coding sequences between human and chimpanzee, [11] found a surprisingly high value of *U *(1.6 per diploid genome per generation). In addition, such approaches, applied to mitochondrial DNA where deleterious mutations predominate, showed that the observed number of non synonymous substitutions fit well with a model in which the strength of selection is exponentially distributed [12].

Mutation (*M*) and selection (*S*) experiments start from a single homozygous individual and measure fitness related traits on derived progenies obtained from (i) random or directed mutagenesis (mutation experiment *M E*); (ii) accumulation of mutations during a large number of generations carried out without directed selection in addition to minimizing the effects of natural selection (mutation accumulation *M A*); (iii) accumulation of mutations during a large number of generations carried out with selection on a particular trait (selection experiment *S*).

Mutation experiments (*M E*) measure the fitness of a set of independently-derived single step mutants evaluated under various environments and provide with direct information on the distribution of mutational effects. Using site-directed mutagenesis on a RNA virus, [13] showed that almost 40% of the mutations were lethal, but found a high proportion of beneficial mutations (4%) that could partly be explained by the chimeric nature of the virus and its poor adaptation to the laboratory conditions [14] demonstrated using *Pseudomonas fluorescens *that, across various environments, the effect of beneficial mutations on fitness is exponentially distributed and characterized by many mutations with small effects and few mutations with large effects. Those results are in accordance with the idea that beneficial mutations are drawn in the right-hand tail of the distribution of mutational effects, so that their distribution belongs to the exponential family, as predicted by the extreme value theory [15]. *M A *experiments consist in deriving single descent lines from one individual in controlled favourable conditions, therefore limiting the effects of natural selection. At the end of the experiment, each new line has accumulated mutations in a neutral fashion, *i.e*. regardless of their possible phenotypic effect. The variance between lines provides with an estimate of the mutational variance [16]. Further hypotheses about the shape of the distribution of mutational effects allow to estimate the genome-wide mutation rate (*U*) and the average fitness effect of a mutation can be inferred from the distribution of fitness-related traits between the *M A*-lines [17]. *M A *experiments have been undertaken in *D. melanogaster *[18-20], *C. elegans *[21-23], *E. coli *[24] and *A. thaliana *[25,26]. Those experiments reveal that mutations alone can generate a considerable amount of phenotypic variability, and drive the derived lines several units of residual standard deviation away from the phenotypic value of the initial homozygous individual. Interestingly, the estimates of the genome-wide mutation rate may vary from several orders of magnitude depending on the species and the trait under consideration (reviewed in [27]). However, one has to be very cautious with such estimates. First, because they may heavily depend on hypotheses about the underlying distribution of the mutational effects, and second, because of possible bias due to statistical artifacts. In particular, there is still a controversy about the proportion of deleterious or slightly deleterious mutations as opposed to advantageous mutations resulting from *M A *experiments [28,29].

Selection experiments (*S*) directly address, for a given trait, the question of the rate of occurrence of beneficial mutations. For instance, [30] observed the occurrence of 66 new advantageous mutations in an experiment of *E. coli *culture over 1000 generations. In divergent selection experiments, the initial inbred is splitted into two populations, that are artificially selected for highest and lowest values of a given trait. The responses to selection in both directions, as well as the differences between high and low populations provide information on the variance created by mutation that can be exploited for selection. Typical outcomes of such experiments are estimates of the so-called mutational heritability, which is the ratio of the input of mutational variance per generation over the residual variance *V*_*m*_/*V*_*E*_. Classically, the slope of the response to selection provides with an estimate of heritability [31]. Because selection experiments start from a fixed material (*i.e*. homozygous at all loci), the only source of genetic variance that can be used by selection comes from new mutations and the mutational heritability can be estimated directly from the slope of the response to selection [32,33]. Divergent selection experiments have been undertaken in *D. melanogaster *[34], mice [35], *C. elegans *[36], and *Chlamydomonas *[37]. The linear response rate is remarkably similar among experiments considering the variety of traits and organisms, and ranges between 0.14 to 0.85 phenotypic standard deviations per generation. Corresponding estimates of mutational heritability falls in the range of 4.10^-4 ^to 7.10^-3 ^(reviewed in [38]). All these experiments demonstrate that the input of new variation through *de novo *mutations is substantial and may explain part of the response to selection during the course of adaptation in natural populations. However there are growing evidence that adaptation also take place from standing genetic variation. One of the most well known examples is the changes in plant architecture during maize domestication. Actually, these changes are governed by a major gene, namely *Tb1 *[39] for which the cultivated allele was found at low frequency in natural populations of teosinte, the wild ancestor of maize [40]. It was shown in a subsequent study by [41] that a number of other domestication-related alleles are present as cryptic variation in teosinte populations. Selection from cryptic variation can have important consequences on the patterns of molecular diversity. In particular, the molecular signature of selection is reduced when the polymorphism pre-exists the selective event and the mutant allele has increased in frequency before selection occurs. In some cases, typical patterns of soft sweep [42], which are hardly distinguishable from patterns of neutral variation, can be obtained. The consequence of soft sweeps is an underestimation of the number of loci contributing to adaptation [43].

Flowering time is a key factor in the adaptation of plants to environmental conditions. During plant development, flowering time determines the end of the vegetative growth, *i.e*. the period during which a plant accumulates resources. It is therefore a key component of the life cycle, that needs to occur at the right climate period. Maize (*Zea mays ssp mays*), a cultivated annual and allogamous species, is a spectacular example of adaptation to an extremely wide range of climatic conditions. During the last century, intensive selection on flowering time has allowed the cultivation of this originally tropical plant in higher latitudes. Southward and Northward crop expansion was made possible by the fixation of alleles favouring flowering in longer days at lower temperatures, and by the elimination of photoperiod sensitivity. In Europe, the range of variation for flowering time between the latest dent lines and the earliest flint lines that are currently used as parents of hybrids is about 25 days. Maize flowering time is primarily determined by the timing of the transition from the vegetative to reproductive phase of the shoot apical meristem [44]. Flowering time is therefore a complex trait and has been extensively studied. Several candidate genes involved in its variation have been identified through QTL metanalysis [45].

In the present study, we were interested in the potential for response to selection of maize inbred lines, which are generally considered as a fixed material when used either for genetic analysis or in selection, but which may encompass some residual standing variation. Our experiment differs slightly from classic SE experiment because as opposed to long-term maintained laboratory strains for model species where conditions can be strictly controlled, we purposely used commercial maize inbred lines seed lots as initial populations. The aim was to document the relative contribution of standing variation and new mutations in the response to selection in this particular material. We undertook two biological replicates of a divergent selection experiment on flowering time starting from 2 maize commercial seed lots considered as inbred lines, an early American flint (*F*252) and a late iodent dent (*MBS*847, thereafter called *MBS*). This selection experiment, starting from a supposedly fixed material, was set up fifteen years ago to (i) characterize the response to selection in two directions, Early and Late flowering; (ii) elucidate the relative role of standing variation versus new mutations in the variability exploited in the response to selection; (iii) better understand the genetic bases of flowering time variation. Surprisingly, a significant response to selection was observed within a very short amount of time (7 generations). Such a fast response is partly determined by the segregation of alleles at a major flowering time QTL in the *F*252 population, but we also found consistent evidence of polygenic variation resulting from new mutations.

## Results

**Figure 1:**
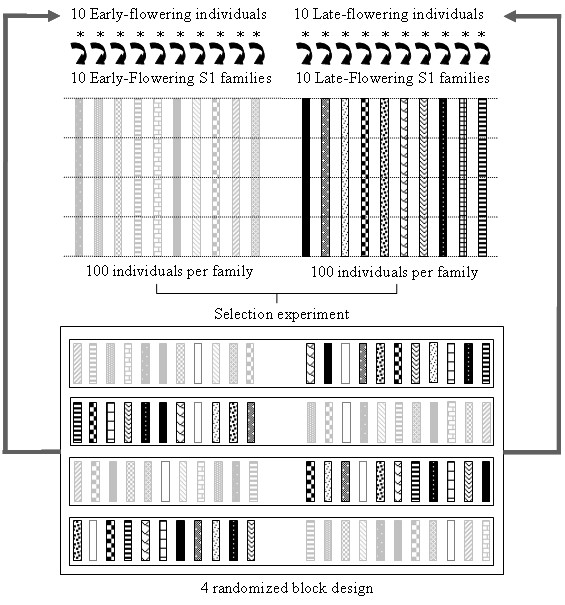
**Description of the Selection Scheme**. Selection was conducted independently for individuals derived from *F*252 and *MBS *in order to obtain an Early and a Late population from each original seed lot. At each generation, S1 families produced by the selfing of the 10 most early-flowering individuals (rectangles with light gray patterns) and the 10 most late-flowering individuals (rectangles with black patterns) of the previous generation are phenotypically evaluated. Hundred individuals per family were sown in a 4 randomized block design. Each block therefore encompasses 25 individuals of each family and contains individuals from the Early and the Late population, as well as a control plot (open rectangles with a dashed line). The same protocol was repeated over generations.

Two maize inbreds *F*252 and *MBS *chosen as initial populations constituted 2 biological replicates of a divergent selection experiment. In each replicate, we selected an early flowering and a late flowering population (Figure 1), called hereafter the Early *F*252, the Early *MBS*, the Late *F*252 and the Late *MBS*. Our selection experiment was set up in order to evaluate the ability of a material supposedly fixed according to maize breeding criteria to respond to selection and to measure the mutational heritability. Below, we first describe the demographic features of our experimental scheme. Second, we report a surprisingly fast response to selection and investigate two alternative hypotheses that may explain it: the fixation of a pre-existing advantageous mutation with major phenotypic effect and the generation of new genetic variability by mutation at loci controlling flowering time.

### Effect of the selection scheme on the effective population size

**Figure 2:**
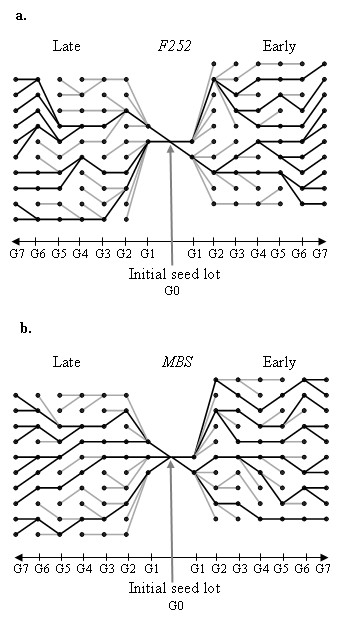
**Genealogies of selected individuals in the four populations**. From the initial seed lots (*G*0), individuals were selected for 7 generations (black dots). An Early and a Late population were derived from *F*252 (a) and *MBS *(b). Genealogies of the individuals of the last generation (*G*7) are indicated in black (individuals connected by black lines). Grey lines indicate the genealogy of individuals that were retained during the selection experiment but that did not contribute to the last generation (*G*7).

In each population, 10 individuals were selected at each generation. In order to limit random genetic drift, an additional constraint on the genealogies was imposed in our selection scheme: we maintained at least two main lineages per population, so that all individuals of the current generation descend from at least two individuals selected in 1993. The Late *MBS *population descends from three individuals in 1993, while the other populations (Early *MBS*, Early and Late *F*252) descend from only two individuals in 1993. Genealogies of the individuals selected in 2001 in all populations are given in Figure 2. From the genealogies of selected individuals, we estimated the effective population size of each population from the variance of the offspring number (10). The effective population size was extremely low during the first two generations, reflecting the strong selection. Since 1998, it became slightly higher than expected in a pure drift model, ranging from 8 to 20 (Table 1).

**Table 1:** Estimate of effective population sizes (*N*_*e*_) and heritabilities (*h*_2_) from generations *G*2 to *G*7 in each four populations

Population Line	Early *F*252		Late *F*252		Early *MBS*		Late *MBS*	
	***N***_***e***_	***h***^**2**^	***N***_***e***_	***h***^**2**^	***N***_***e***_	***h***^**2**^	***N***_***e***_	***h***^**2**^
*G*2	3.1	0.28	6.8	**0.61**	5.8	*0.14*	13.5	**0.68**
*G*3	10.1	0.50	20.2	**0.74**	10.1	-*0.20*	13.5	**0.62**
*G*4	13.5	0.43	10.1	**0.78**	13.5	0.34	13.5	**0.65**
*G*5	13.5	0.31	13.5	**0.85**	10.1	-*0.10*	12.5	**0.48**
*G*6	20.2	**0.46**	13.5	**0.87**	10.1	*0.07*	10.1	0.27
*G*7	8.1	0.30	8.1	**0.81**	8.1	0.35	8.1	*0.13*

Effective population sizes were computed from the number of offsprings (see equation 10). Heritabilities were computed as the ratio of the genetic variance between genotypes of the same population over the total phenotypic variance (between genotypes + within genotypes). Phenotypic values were obtained from *S*2 progenies of each genotype evaluated in 2004 and 2005 (see Material and Methods). For the heritabilities, the character typing indicate the level of significance of the genetic variance: *italic *= non significant, normal = ≤ 5%, **bold **= ≤ 10^-3^.

### Response to selection

Phenotypic data collected on selfing progenies of all selected individuals of the Early *F*252, Late *F*252, Early *MBS *and Late *MBS *were analysed in an 2-year evaluation trial along with their initial inbred lines *F*252 and *MBS*. On average, flowering time for the control line *F*252 was 22 days, with a residual standard deviation of 0.90. For the control line *MBS*, the average flowering time was 35 days, with a residual standard deviation of 0.56. Controls were further used to correct all phenotypic data for field heterogeneity. An ANOVA on corrected phenotypic values was performed separately for the individuals derived from each inbred line to test for the effects of population (Early vs Late), year of selection (from *G*1 to *G*7), and genotype within population and year of selection (see (1)). For both *F*252 and *MBS *derived populations, all the effects were highly significant, including the population by year of selection interaction (data not shown).

**Figure 3:**
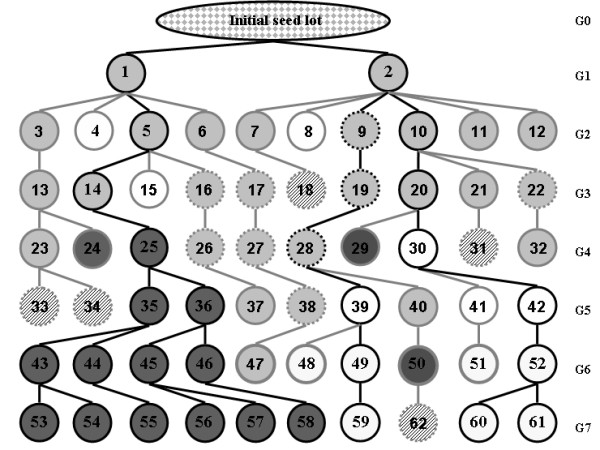
**Genealogy of the genotypes at the QCK5e06 locus in the Late *F*252 population**. Generations are numbered from *G*0 to *G*7. Circles represent the individuals numbered from 1 to 61. They are coloured according to their genotype at locus QCK5e06: light grey = heterozygotes, dark grey = homozygotes for the late allele, white = homozygotes for the other allele. Residual heterozygosity in the initial seed lot is represented by a grey/white pattern. Out of 31 individuals genotypes from the initial seed lot, only one was heterozygote, and all the others were homozygotes for the white allele. Dashed lines around circles indicate missing genotypic data. The genotypes of the corresponding individuals were inferred from their progenies as described in the material and methods (except for individuals 18,31,33,34 and 62 which were treated as missing data). Phenotypic information for individuals 27, 28 and 40 was missing. They were attributed a genotypic value for flowering time by averaging the genotypic values of the individuals of the same sub-family at the same generation (23, 24, 25, 26 for individual 27; 29, 30, 31, 32 for individual 28; and 39, 41, 42 for individual 40).

For *F*252, flowering time ranges from 20 to 34. Genotypic values of the Early *F*252 range from 20 to 24. Its mode is 22 and corresponds to the average flowering time of the control line. In contrast, genotypic values for the Late *F*252 exhibit a much wider range of variation, from 21 to 34. Actually, the genotypic distribution of flowering time in the Late *F*252 can be pictured as a mixture of two overlapping distributions, with some individuals characterized by a very late flowering time. Tracing back the genealogy of those very late individuals (Figure 3), it appears that they all belong to a same subfamilly, with the individuals of the last generation sharing a single ancestor at generation *G*4 (individual #25 in Figure 3). Based on this observation, we decided to separate the Late *F*252 into two different populations, namely the Late-VL *F*252 (VL = Very Late) comprising the individuals descending from individual #25, and the Late-NVL *F*252(NVL = Not Very Late) comprising the rest of the population. For the Late-NVL *F*252, flowering time ranges from 21 to 29, with a mode of 24. For the Late-VL *F*252, flowering time ranges from 24 to 34, and the distribution is uniform. For *MBS*, flowering time ranges from 33 to 36 days in the Early *MBS*, and from 35 to 40 days in the Late *MBS*. The mode of each distribution are 35 and 38, respectively. Again, the mode of the distribution of the Early *MBS *is close to the average flowering time of the *MBS *control line.

**Figure 4:**
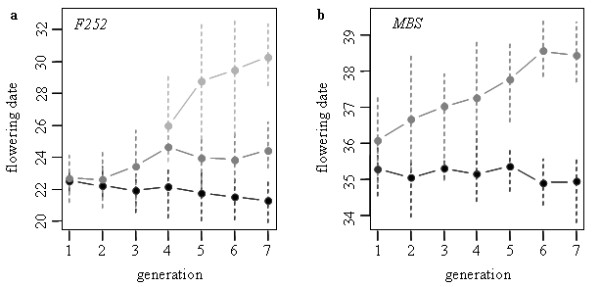
**Response to selection from generations *G*1 to *G*7 in the *F*252 (a) and *MBS *(b) populations**. Flowering time was measured on *S*2 families for each genotype of the genealogies in a two years evaluation trial (2004 and 2005). For each population, the average genotypic values (circles) and the interval between the extremes genotypic values (dashed bars) are plotted against the number of generations. Black and Grey colors represent the Early and the Late population respectively. In the Late *F*252 population, we estimated genotypic values separately for the Late-NVL *F*252 (dark-grey) and the Late-VL *F*252 (light-grey). The response to selection is significant in all populations except the Early *MBS *(see Table 2). For example in *G*7, the Late *MBS *flowers on average 3 days later than both the initial seed lot (not shown) and the Early *MBS *population. Note that the Late-VL *F*252 genotypes at generation *G*6 and *G*7, all descend from a single individual at generation *G*4 (Figure 3).

The total range of variation for flowering time was 15 standard deviations for *MBS *and 12 standard deviations for *F*252. This observation is indicative of a fast response to selection. Consistently, we pointed out a significant difference between Early and Late populations in the ANOVA. In addition, the significant effect of the population by year interaction in both lines reveals that at least one of the 2 populations responded to selection. In order to quantify this response, a regression analysis of flowering time over the generation of selection (3) was performed separately in each population derived from each line. We performed two separate analyses for *F*252, one with all genotypic values, and another one after discarding the Late-VL *F*252. Results are reported in Table 2. A significant response to selection is observed in all populations, excepted the Early *MBS*. Interestingly, this response is stronger in the Late populations than in the Early populations. This asymmetry is well illustrated in Figure 4. When Late-VL *F*252 individuals were discarded from the analysis of the Late *F*252, the average response to selection in the Late-NVL *F*252 and the Late *MBS *is of the same order of magnitude (0.34 days per year for Late-NVL *F*252 and 0.40 days per year for the Late *MBS*, Figure 4 and Table 2). By contrast, when considering all individuals in the Late *F*252 population, the observed response to selection was much stronger (1 day per year), suggesting the existence of a polymorphic locus with a major effect on flowering time. Finally, Figure 4 also illustrates the linearity of the response to selection through time, despite the low effective population sizes, and a short time frame (7 generations).

**Table 2:** Response to selection and estimates of initial () and mutational () heritabilities in the four populations

Population	Estimated parameters	Late-NVL *F*252	Early *F*252	Late *MBS*	Early *MBS*
		28.35	21.31	38.45	34.95
*R*^*b*^		0.34***	- 0.18***	0.40***	- 0.04^*ns*^
*R *s.e.^*c*^		0.081	0.044	0.039	0.030

Model 1	* 10^-2^	2.9 (1.3-6.1)	1.4 (0.7-2.9)	3.3 (1.9-6.9)	0.3 (0.0-0.8)
Model 2	* 10^-2^	20.8 (9.7-44.0)	12.5 (5.8-25.8)	28.4 (16.5-56.8)	2.4 (0.0-6.4)
Model 3	* 10^-2^	1.2	0.5	4.8	*ns*
	* 10^-2^	2.5 (1.03-5.79)	1.3 (0.5-2.8)	1.9 (0.49-5.36)	*ns*

^*a *^Average genotypic value at *G*7. ^*b *^Response to selection as estimated using (3). Significance is indicated (*** = *p *< 0.001, *ns *= non significant). ^*c *^standard error of *R*.

Heritabilities are computed from the average response to selection as the ratio of the additive genetic variance to the residual variance by using an EM algorithm (11 and 9). In parentheses are the 95% confidence intervals that were simulated by taking into account uncertainty in the estimated response to selection and in the residual variance,  (see Material and Methods). Genetic variance is assumed to come either from new mutations (Model 1,  = 0) or from standing variation (Model 2,  = 0) or else from both (Model 3). In Model 3, the value for  corresponds to the minimum value which gives a non zero estimate of the mutational heritability.

Altogether, these results called for further investigation within the Late *F*252, in order to better understand the genetic bases of the differentiation between the Late-NVL *F*252 and the Late-VL *F*252. We therefore searched for polymorphism at ten candidate loci for flowering time using RFLP markers, and found one that segregates within the Late *F*252. Apart from the discontinuity caused by this Late-VL *F*252 within the Late *F*252, the linear response to selection in all populations clearly suggests a polygenic basis for flowering time, which we further analysed by estimating the mutational heritability.

### Validation of a candidate locus by association mapping

**Figure 5:**
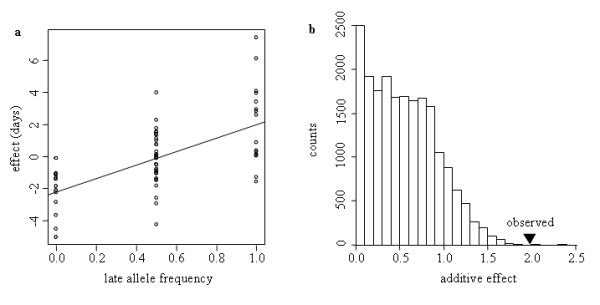
**Association between the polymorphism at the QCK5e06 locus and flowering time variation in the Late *F*252**. (a) Phenotypic effect associated with the frequency of the Late allele. Datapoints represent the flowering time deviation from the average genotypic value for each genotype in the genealogy as a function of the frequency of the Late allele at the QCK5e06 locus. The straight line represents the slope of the regression. (b) The simulated distribution of estimated additive effect for flowering time, *a*, was obtained by simulating a matrix of genotypes by gene dropping and performing an association test with the corresponding observed phenotypic matrix (see Material and Methods). Resulting *a *values from 20,000 simulations are plotted. The position of the triangle indicates the observed additive effect associated with the polymorphism at locus QCK5e06.

We searched for polymorphism at ten candidate loci for flowering time using RFLP markers. Markers were developed from 8 cDNA probes and 2 additional probes obtained by PCR reaction on candidate genes *Zf l*1 and *Zf l*2. As shown in Table 3, among the 8 cDNA probes tested, some of them (including *Zf l*1 and *Zf l*2 not shown in Table 3) had fixed different alleles between populations issued from one parent (*F*252) or the other (*MBS*), others were monomorphic, and one probe, namely QCK5e06, had fixed different alleles between the Early *F*252 and the Late *F*252. This probe was also polymorphic within the Late *F*252 population. We therefore decided to perform some additional analyses with QCK5e06 in the Late *F*252. First, we genotyped *n *= 31 individuals of the initial seed lot using the same RFLP procedure as described above, and we found one heterozygote at the QCK5e06 locus. The frequency *p *of residual heterozygosity was therefore estimated as *p *= 0.032. Second, we genotyped 4 offsprings of each individual of the genealogy. The resulting genotypes and genotypic values obtained for 61 individuals (Figure 3) were used in an association mapping analysis to estimate the additivity *a*_*obs *_and the dominance *d*_*obs *_as described in (12). We found *a*_*obs *_= - 1.96 while *d*_*obs *_value was close to zero (-0.12) suggesting that the additive effect of the gene is fairly strong as compared to the dominance (Figure 5.a). The model explained 35% of the phenotypic variation. We addressed the following question: Is the QCK5e06 locus involved in the phenotypic variation for flowering time in our divergent selection experiment ? We compared our observed values of *a *and *d *to distributions generated for both the additivity and the dominance under the null hypothesis, H0, of random segregation of alleles at this locus in the observed genealogy starting from 2 heterozygotes in 1993 (Figure 3). As shown in Figure 5.b, only 2 out of 20,000 simulations performed under H0 gave an *a *value above *a*_*obs *_suggesting that the polymorphism at the QCK5e06 locus is associated with phenotypic variation for flowering time. In contrast, for the dominance effect, it was not possible to reject H0 (data not shown). Therefore the effect of the QCK5e06 is mainly additive: the average flowering time in the Late *F*252 computed across all generations was 23 and 27 for both homozygotes, respectively, and 25 for the heterozygotes. Finally, we asked whether this association resulted from selection at this locus or whether it resulted from random drift in the genealogy. In the Late *F*252, the frequency of the late allele increased from *f*_0 _= 0.016 (*p/*2) in the initial seed lot to *f*_7 _= 6/9 = 0.67 at generation *G*7 (Figure 3). We estimated the effect of drift on changes in allelic frequencies by simulating the allelic frequency distribution in a theoretical population with the same effective population sizes at each generation than the observed values in the Late *F*252 (equation 10 and Table 1). From the distribution obtained with 10,000 simulations, the probability of observing *f*_7 _≥ 0.67 starting from *f*_0 _= 0.016 is below 1%. Therefore, the observed association is unlikely to result from drift alone.

**Table 3:** Description of probes and RFLP genotyping

Probe name	GeneBank accession	Linkage group	Bin	**Map coordinate**^***a***^	RFLP result EcoR1 digest	RFLP result Mbo1 digest
QCI22a07	CF041118	3	3.01-3.02	26.7	fixed differences^*b*^	fixed differences^*b*^
QBI1h05	CF006474	4	4.06-4.07	180.2	fixed differences^*b*^	fixed differences^*b*^
QCO39e08	CF057945	5	5.03-5.04	169.6	monomorphic^*d*^	fixed differences^*b*^
QCK5e06	CF045980	6	6.01-6.02	44.2	polymorphic^*c*^	monomorphic^*d*^
QAI1a06	CD981043	6	6.02-6.04	65.7	fixed differences^*b*^	fixed differences^*b*^
QCK7b02	CF047313	6	6.04-6.05	129.1	fixed differences^*b*^	monomorphic^*d*^
QCG39e03	CX129553	6	6.04-6.05	110.6	monomorphic^*d*^	monomorphic^*d*^
QCC6e03	CX129533	9	9.03	99.4	monomorphic^*d*^	monomorphic^*d*^

^*a *^Map coordinates on IBMconsensus Gnp2004 (Falque et al, 2005). ^*b *^Populations derived from *F*252 and populations derived from *MBS *are monomorphic but are fixed respectively for different alleles. ^*c *^Two alleles segregate at least within one of the four populations. ^*d *^Only one allele was detected among all offsprings analysed.

### Mutational heritability and evolution of genetic variation

The observed linear response to selection in all populations (excepted the Early *MBS*) implies the existence of genetic variation within each population at each generation. We used the phenotypic evaluation trial to estimate the genetic variance between selected individuals at each generation in each population. The resulting within population heritabilities estimates computed as in (2) are given in Table 1. They were significant at almost all generations for both lines, except in the Early *MBS*, and all the values were surprisingly high. As expected from the asymmetry of the response to selection (figure 4.a and 4.b), the within population heritabilities were higher in the Late than in the Early populations. They were comprised between 0.13 and 0.81 in the two Late populations (Table 1), meaning that up to eighty percent of the phenotypic variation for flowering time is genetically determined. Finally, the patterns of variation of the within population heritabilities correlate with the patterns of the response to selection. For example, the strong response observed during the first 4 generations of selection in the Late *F*252 population (Figure 4.a) can be explained by correspondingly high values of within population heritabilities (Table 1). At the opposite, the apparent lack of genetic variability at generation 6 in the Early and the Late *MBS *(Table 1) might be partly responsible for the poor response to selection between generations 6 and 7 (Figure 4).

Starting from a supposedly fixed material (commercial inbred lines), we observed a significant genetic variation at each generation within three out of the four populations, namely the Late-NVL *F*252, the Early *F*252 and the Late *MBS*. This motivated us to quantify the input of new variation required to explain the associated response to selection for flowering time, *i.e*. mutational heritability. We therefore modelled the response to selection expected under an infinitesimal model with or without mutations, but taking random genetic drift into account. The upper bound for the mutational heritability was provided by a model in which all the observed variation is brought by mutations that occurred during our selection experiment (Model 1), *i.e*.  = 0. Resulting estimates, ranging from 0.003 to 0.033 (Table 2) are much higher than what is generally found in the literature (see section **Introduction**). The lower bound for the mutational heritability is  = 0, *i.e*. all the observed variation is coming from standing variation (Model 2). Finally, we assumed a non zero mutational heritability and estimated the upper bound of the initial genetic variance (Model 3). This gave us three different estimates for the pair (, ). The results are given in Table 2. They are in good agreement with previous observations: under Model 2, high values of the mutational heritability were obtained for the populations that best responded to selection (0.025 and 0.019 for the Late *F*252 and the Late *MBS *respectively), and low values for the Early *F*252 (0.013). Notice the similarities between the estimates of mutational heritabilities in the Late populations of the two biological replicate experiments, *F*252 and *MBS*.

**Figure 6:**
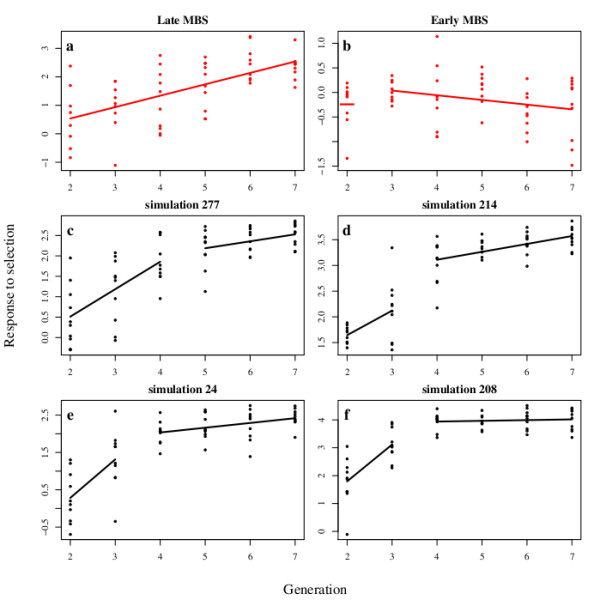
**Segmented regression model with one breakpoint that best fit observed and simulated data**. Segmented regression on observed data from the Late *MBS *population (a) and the Early *MBS *population (b). (a) Late *MBS *population: the best model is a single line (breakpoint occurs at the generation 7 or later). (b) Early *MBS *population: the best model is provided by a breakpoint at generation 2 leading to a point at this generation and a line between the generations 3 to 7. Examples of segmented regression on simulations of the selection experiment (c to f). The selection experiment was simulated with the average initial heritability = 0.02841 estimated from the observed data of the Late *MBS *population with Model 2 (no input of *de novo *mutation see text for the details), and *n*_*P *_= 100 and *n*_*H *_= 60. The four examples were chosen among those that display the same average response to selection over the seven generations. (c) The best model is provided by a breakpoint at generation 4 leading to two segments, the first between generations 2 to 4 and the second between generations 5 to 7. (d to f) The best models are provided by a breakpoint at generation 3 leading to two segments, the first between generations 2 to 3 and the second between generations 4 to 7. In (f), the second segment is horizontal and corresponds to a plateau.

In order to check whether the response to selection could be attributed solely to standing genetic variation, we simulated possible outcomes of the selection experiment without considering the input of *de novo *mutations (Model 2). In our simulations, we considered an initial population with *n*_*P *_loci segregating, of which *n*_*H *_are heterozygous and (*n*_*P *_-* n*_*H*_) are fixed differences between the individuals. Allelic effects were drawn in an exponential distribution to allow for unequal gene effects. They were further scaled to match the value of the initial heritability  estimated from the experimental populations. Starting with a null mutational heritability, we observed a rapid exhaustion of the initial genetic variability leading to a decrease of the rate of the response to selection through time (Figure 6-b to 6-e). The response to selection was therefore non-linear. In contrast, the response to selection in the experimental populations is linear through time (Late-NVL *F*252, Early *F*252, Late *MBS*, Figure 6-a) or increases after *G*2 (Early *MBS*, Figure 6-b).

**Figure 7:**
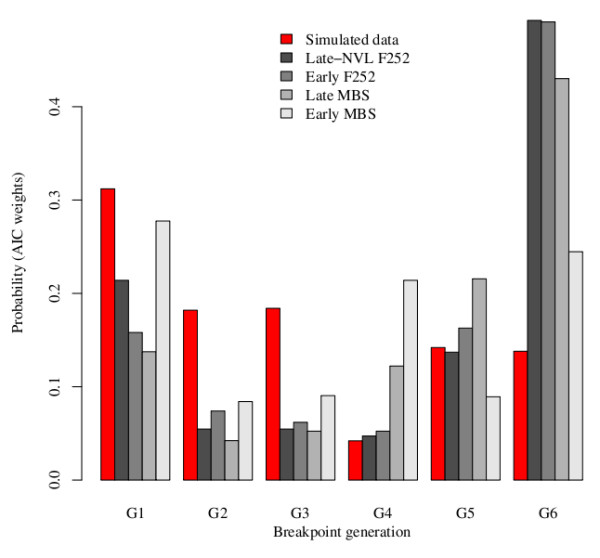
**Distribution of the AICc weights of segmented regression models with one breakpoint in simulated and observed data from the four populations**. The selection experiment was simulated with the same parameters ans in Figure 6. For each simulation, segmented regression with a single breakpoint at each of the seven generations were fitted. The red bars represent the proportion of simulations in which the best fitted model corresponds to a breakpoint at the given generation. Similar results were obtained across simulations with the initial conditions indicated in Table 4. The grey bars represent the AICc weights computed by fitting segmented regression with a single breakpoint at each of the seven generations on the observed data of the two *F*252 (Early and Late Not Very Late) and *MBS *(Early and Late) experimental populations. These weights give the probability that the change in the rate of the response to selection occurred at generation *G*_*i *_(2 ≤ *i *≤ 7). Note that while in the simulations a higher probability is associated with a breakpoint occuring at *G*1, 3 out of the 4 experimental populations are consistent with a linear response to selection through time, *i.e*. breakpoint at *G*7.

In order to provide statistical support to these observations, we applied a linear segmentation regression [46] on simulated data, as well as on experimental data. The rationale of this approach was the following: the segmentation model supposed a single breakpoint in the rate of response to selection between *G*2 and *G*7; applied to the observed responses to selection in the experiment, the segmentation model estimated the probability that the breakpoint occurs at *G*2, *G*3, ... or *G*7. These probabilities were compared to the number of breakpoints occurrences at *G*2, *G*3, ... or *G*7 in the simulated data. As illustrated in Figure 7, the probability distribution under the null hypothesis of no mutation differed from the probability distributions of the observed data. This was true for all sets of initial conditions (Table 4). In particular, the probability that simulated data exhibited a linear response to selection was low.

**Table 4:** Monte-Carlo simulations of the response to selection under the null hypothesis of absence of de novo mutations

***n***_***P***_	***n***_***H***_	**estimates**	**other**^***a***^	% simulations with linear response	**Average response**^***b***^	*P value*
100	100	Late MBS	add	0.08	0.67 (0.43-0.99)	0.019*
100	60	Late MBS	add	0.12	0.46 (0.25-0.74)	0.320^*ns*^
100	60	Late MBS	dom	0.28	0.49 (0.23-0.76)	0.317^*ns*^
100	60	Early F252	add	0.24	-0.35 (-0.57 - -0.16)	0.068^*ns*^
100	20	Late MBS	add	0.33	0.22 (0.08-0.38)	0.046*
100	10	Late MBS	add	0.51	0.13 (-0.02-0.28)	0.006**
20	20	Late MBS	add	0.06	0.49 (0.27-0.78)	0.288^*ns*^
20	10	Late MBS	add	0.18	0.31 (0.13-0.55)	0.220^*ns*^
20	5	Late MBS	add	0.32	0.18 (0.04-0.36)	0.030*

^*a *^add = additivity within and between loci. dom = dominance of the most favourable allele. ^*b *^95% confidence interval is given between brackets.

Each simulation is defined by an initial number of polymorphic loci *n*_*P*_, an initial number of heterozygous loci *n*_*H *_and the experimental population from which the initial genetic variance was estimated. Corresponding values of initial heritabilities  are given in (Table 3). % simulations with linear response is the fraction of the 500 runs for which the model that minimizes *AICc *in the segmented regression is the one with a breakpoint at *G*7 or after, indicating a linear response to selection. Average response is the average response to selection computed as in (3) for each run and averaged over the 500 runs. *P value *contains the percentage of simulations for which the average response to selection is lower than the one observed in the corresponding experimental population. When *n*_*H *_is too high, the simulated response to selection is always higher than the observed one. When *n*_*H *_is too low, the simulated response to selection is always lower than the observed one.

Simulated data rather pointed out to a breakpoint at *G*2, suggesting that standing genetic variation was exhausted with a greater probability after 2 generations of selection. The only exception was the Early *MBS *population, where the best segmentation model was the occurrence of a breakpoint at *G*2, consistent with the lack of significance of the response to selection in that population (Table 2). Note that if qualitative response to selection differed markedly between simulated and observed data, in many case, the average simulated response to selection was not quantitatively different from the observed response to selection. Exceptions were simulations with (*n*_*P *_= 100, *n*_*H *_= 100), (*n*_*P *_= 100, *n*_*H *_= 10) and (*n*_*P *_= 20, *n*_*H *_= 5), which had to be excluded from the analysis because the response to selection was either too high or too small as compared to the experimental one. When the number of heterozygous loci in the initial population increased, the percentage of simulations exhibiting a linear response decreased, and the average rate of response tended to increase (Table 4). Overall, three lines of arguments support the hypothesis that new mutations have contributed to the observed response to selection: (i) in the absence of new mutations, the response observed in the experiments could not be reproduced without supposing a high and therefore unlikely number of polymorphisms in the initial population; (ii) in the subsample of simulations that displayed a quantitative response to selection similar to the observed response, non-linearities were observed in more than 75% of the simulations; (iii) the pattern associated with genetic variance fluctuations over time differed between the simulations and our observed data (genetic variance decreased in the simulations (not shown) and stayed constant in the experiment (see above)).

## Discussion

Our study reports an original selection experiment conducted on a crop in open field conditions. By setting up this experiment, we aimed at investigating the rate and limits of the response to selection from a highly inbred genetic material. After only 7 generations of selection, we observed a spectacular response to selection and identified a mutation with a major late flowering effect.

The selection protocol (Figure 1) was applied to two different initial inbred lines, *F*252 and *MBS*, which constituted two biological replicates of the same experiment. Commercial seed lots of *F*252 and *MBS *were taken as the initial populations. In maize, the classical breeding strategy consists in searching for new genetic combinations in the progenies of *F *1 hybrids between already existing inbreds [47]. New inbred lines are obtained after several generations of selfing (9 to 12) from the *F *1 hybrid. The seed lots that were used here were taken from inbreds that were maintained for about 10 years since their first registration. They may encompass residual heterozygosity or fixed differences at some loci, either for alleles that were present in the initial hybrid, or for new alleles generated by mutations during the selfing stages. *De novo *variability altering gene expression has been observed within inbred stocks of mice maintained over 200 generations of brother-sister mating [48].

The selection experiment consisted in splitting each initial seed lot into two populations, one selected for earliness, and the other selected for lateness. In the Early population, the 10 earliest individuals were selected and selfed to produce the next generation; in the Late population, the 10 latest individuals were selected and selfed to produce the next generation. Because of the selfing process, no recombination occurs between selected individuals, and each of the 4 resulting populations consisted in a set of independent lines. Note that because of possible contamination in open field conditions, we applied a rigorous protocol for selfing leading to a contamination rate below 0.001. The fact that we did not detect inconsistency between the phenotypes of the individuals and their genealogy strengthens the idea that cross contamination has not occurred between populations.

**Figure 8:**
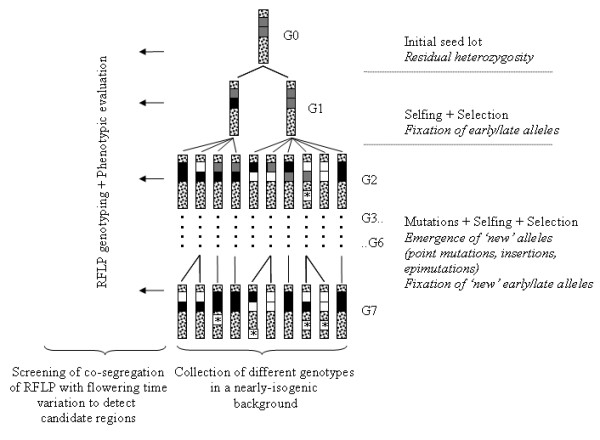
**Experimental procedure with a special emphasize on the evolutionary processes that operate through generations**. Bars represent the diploid genotypes present in a virtual population (Early or Late population derived from either *F*252 or *MBS*). White and black boxes indicate homozygous regions that are fixed for one of the allele (Early or Late). Heterozygosity can result either from standing variation present at *G*0 (grey box) or from new mutations occurring in the subsequent generations (white box with a star). Mutations from both sources can become fixed during the course of the experiment (*G*1 to *G*7) because of genetic drift and/or selection. At each generation, molecular polymorphism (as revealed by RFLP on 10 candidate regions) and phenotypic variation were evaluated in order to detect association between RFLP and flowering time variation.

We observed a fast response to selection between Early and Late populations in both replicated experiments. Such a fast response to selection from nearly fixed inbred material can be explained either by the existence of heritable genetic variation in the initial populations, or by the generation of heritable genetic variation through *de novo *(epi)mutations (Figure 8). While we expected *de novo *mutations to be the main cause of the response to selection, we found several lines of evidence suggesting the importance of initial genetic variation in this experiment. A striking feature of the Late *F*252 is the occurrence of very late individuals (Late-VL *F*252) that all descend from a single individual at *G*4 (Figure 3). Actually, 35% of the phenotypic variation within the Late *F*252 population is explained by the segregation of 2 alleles at the QCK5e06 locus which were both present in the initial seed lot. The RFLP probe QCK5e06 which was found polymorphic within the *F*252 initial seed lot, with one allele associated with the 'very late flowering' phenotype in the genealogy of the Late *F*252 population (Figure 3) was designed from a maize cDNA library [49] and correspond to a candidate gene for flowering time in maize [45]. Up to now, only a few mutations affecting flowering time have been identified in maize, and mostly confer an early flowering phenotype. *Id1*, which encodes a zinc finger transcription factor, was cloned from a mutation leading to a lack of conversion of the apical meristem from a vegetative to a reproductive state [50]. Two other mutants, *dlf1 *and *lfy1 *have shown specific but weak effect on floral transition, and the *epc *mutation reduces the duration of the juvenile vegetative phase without any effect on the number of leaves in the adult plant [51]. Additionally, the *vgt *mutations [52,53] strongly reduce the number of nodes, indicating that they affect flowering time by accelerating the vegetative to reproductive differentiation of the shoot apical meristem. The *vgt1 *mutation was identified as a 2 kb non coding region positioned 70 kb upstream of an *Ap2 *-like transcription factor shown to be involved in flowering-time control [54]. Finally, *Dwarf8*, a gene identified from a mutant and involved in the gibberellin pathway, was the first gene to be found statistically associated to flowering time variation in a panel of around 100 American inbred lines [55]. However, more recent analyses on broader maize panels revealed much lower and border-line significance [56]. We are currently trying to validate the implication of the QCK5e06 locus in the genetic architecture of maize flowering time. Preliminary results suggest that the delayed development of the very Late *F*252 genotypes is due to a greater number of nodes accompanied by a delayed reproductive differentiation of the shoot apical meristem.

Genetic polymorphism at the QCK5e06 locus only explains part of the response to selection in the Late *F*252 population. Once the individuals of the Late *F*252 that were homozygous for the 'late' allele at the QCK5e06 locus (Late-VL *F*252) were discarded, we observed a linear response to selection in three out of the four populations (Figure 4, Table 2). Responses to selection in the two replicated experiments are remarkably similar. A strong linear response to selection of 0.40 days per year for the Late *MBS *and 0.34 days per year for the Late-NVL *F*252 (Table 2) corroborates significant heritabilities found within populations (Table 1). In the Early populations the response is limited and significant only in the Early *F*252 (decrease of 0.2 days per year - Table 2). We found significant genetic variance for flowering time within each population in the generations *G*2 and *G*3 of Early *F*252 and Early *MBS *replicates (Table 1), consistent with pre-existing variability. Finally, the analysis of the response to selection led to estimates of initial heritability at *G*0 comprised between 2.4% and 14.1% of the residual variance (Table 2). Altogether, this indicates that a maize inbred line, which is generally considered by breeders as a fixed material, remains partially polymorphic, at least at loci involved in the determinism of flowering time. One possible explanation is that heterozygosity has been maintained from the initial *F*252 inbred by natural selection because heterozygosity at some loci confers a selective advantage over homozygosity, a phenomenon called heterosis. While heterosis is known to be important for many traits including flowering time determinism, it is worthwhile noticing that the effect of the QCK5e06 is mainly additive (Figure 5-a) which does not support the maintenance of polymorphism at this locus by heterosis.

The consistency of the response to selection during seven generations cannot be explained solely by residual polymorphism in the initial seed lots but requires the input of new genetic variance by mutation (Figure 8). Indeed, we have shown that a model considering standing variation as the unique source of genetic variation was unlikely to produce a sustained response to selection during 7 generation given the importance of random genetic drift, *i.e*. small effective population size, in our experiment (Figure 7). While this is not a formal proof, it strongly suggests that new mutations have contributed to the observed response to selection in 3 out of the 4 populations (Figure 6). We are currently analysing the genetic polymorphisms within the populations to quantify precisely this contribution. Estimates of the mutational variance ranged between 1.3.10^-2 ^to 2.5^-2 ^units of residual variance per generation (Table 2), and stand as higher bound to what was previously described in other species [38]. In regards with the small effective population size in our experiment, which ranges between *N*_*e *_= 3 and *N*_*e *_= 20, the efficiency of selection likely results from a high mutation rate combined with large effects of beneficial mutations. A high mutation rate is consistent with the complexity of flowering time genetic determinism, with many possible targets for selection [57]. Indeed, the more the number of genes that determine a trait, the more the number of potential targets for beneficial mutations. Our experiment also strongly support the idea that beneficial mutations that are primarily fixed have large effects [58]. Indeed, we found a major allele at the QCK5e06 that explains up to 35% of the phenotypic variation. While population size necessarily constrains the adaptive potential and the dynamics of adaptation, theoretical results suggest that selection within small populations can increase the rate of fixation of advantageous mutations [59,60]. In our experimental scheme, if by chance an advantageous mutation occurs in a genetic background of an individual retained by selection, its initial frequency will immediately raise to 5% (1 heterozygote among 10 individuals), therefore increasing its chance to become fixed. This may explain the fast response to selection observed in our experiment. Overall, our results therefore constitute an experimental evidence for the adaptive potential of small, highly consanguineous populations.

A striking feature of our selection experiment is the asymmetry of the response to selection, characterized by a higher response in the Late populations than in the Early populations. Such asymmetry in the response to selection is classically observed for quantitative traits [61], and can be attributed to epistasis: whenever antagonistic or 'less than additive' epistatic interactions predominate, the response to selection in one direction is predicted to be much easier than in the other direction [62]. Along the same line, we expect a diminishing return of mutation effect in the Early populations and conversely mutations of high effect in the Late populations [63,64]. The 2 initial inbreds *F*252 and *MBS *have been intensively selected for earliness [56,65]. Any new allelic variant therefore occurs within an 'early' genetic background, which may either constraint or accentuate its effect on the phenotype, depending on the direction of selection. The asymmetry of the response to selection associated with a stronger response to selection in the Late populations in both replicates (*F*252 and *MBS*) suggests that beneficial mutation effects are stronger when selecting for lateness in such an early genetic background. Because both initial inbreds were early flowering, and therefore maladapted considering that the target of selection is late flowering, this observation supports previous findings in viruses that small maladapted populations are characterized by higher fixation rates of beneficial mutations [64].

## Conclusions

Our experiment demonstrates that starting from a highly inbred material, it is possible within a few generations to create maize populations that strikingly differ from the initial seed lot for flowering time while preserving most of the phenotypic characteristics of the initial inbred. Such material is unique for studying the dynamic of the response to selection and its genetic determinants. We found that, in addition to the fixation of a standing beneficial mutation associated with a large phenotypic effect, a constant input of genetic variance by new mutations has likely contributed to a linear response to selection over generations. Elevated values of the estimated mutational variance suggest a high mutation rate consistent with the complex genetic determinism of flowering time. Overall, our results provide a glimpse on the adaptive potential of extremely small populations, which may contribute to their persistence in natural conditions.

## Methods

Since the starting of our selection experiment in 1993, the selection procedure has undergone several minor changes (in 1997 and 1998). Below we describe these changes as well as the ongoing procedure. The selection scheme is presented in Figure 1.

### Initial inbred lines

The initial populations were certified base seed lots from two maize inbred lines: an american flint, *F*252, and a late iodent dent, *MBS*847 obtained in 1992 from the breeding companies Agri-Obtention for *F*252 and Mike Brayton Seeds for *MBS*847. *F*252 was first registered in 1979, and *MBS*847 in 1982. In maize, inbreds are obtained from *F *1 hybrids after several generations (6 to 8) of selfing and selection. At each generation, the inbred line is represented by the selfing progeny of a single individual of the previous generation (ear to row) and selfing is done manually to avoid outcrossing. The last generation consists in producing the pre-base seed lot by harvesting all the seeds from the selfing progenies of the selected individual. The pre-base seed lot is submitted to controls for homogeneity before registration. Commercial base seed lots are produced by crossing together pre-base individuals in plots isolated from other maize culture to avoid cross-contamination. In France, the homogeneity and stability of base seed lots is controlled and certified by SOC http://www.gnis.fr/. If necessary, the pre-base stock is renewed by ear to row self pollination using the same protocol as for the production of the first pre-base seed lot. Because our experiment started in 1993, long after the first registration of the inbred lines, it is reasonable to consider that the base seed lots that we used had undergone at least 5 generations of multiplication from the initial pre-base. Therefore, the initial populations resulted from at least 12 generations of selfing. Without mutation, the residual heterozygosity of one pre-base individual is expected to be 1/2^12 ^= 0.00024. We also expect polymorphisms between pre-base individuals which may result either in fixed differences or in residual heteozygosity in the base seed lot. Each line was treated separately as an independent biological replicate of the selection experiment. Notice that these are not true replicates, and differences in response among selection lines can be due to differences in their genetic features, as well as in differences in the stochastic events associated with mutation, drift and selection.

### Divergent selection experiment

All the experiments took place at Gif sur Yvette in France. The pedigree of the selected individuals was recorded since the beginning of the experiment.

1993: For each line (*F*252 and *MBS*), about 60 plants of the initial seed lot were sown. Female flowering time was recorded, all the individuals were selfed and kernels were harvested. The selfing progenies of the three earliest individuals constituted the three families of the Early population. The progenies of the three latest individuals constituted the three families of the Late population. Seeds were stored at +6°C in a cool chamber.

1997: Selfing progenies of the plants selected in 1993 were grown in a randomized block design. For each family, four rows of 25 plants were sown together at a density of 25000 plants/ha. Spacing between rows was 80 cm. During flowering period, selfing was performed on each plant as soon as both male and female flowering occurred. The selfing date was recorded in days after July 1st, kernels were harvested and weighted (basal kernel for each plant). In the Late populations, among the latest individuals, we selected 10 with the highest kernel weight. In the Early populations, among the earliest individuals, we selected 10 with the highest kernel weight. All the seeds were stored at 6°C in a cool chamber.

1998 and following years: In order to better control the environmental effect, a randomized block design was set up (Figure 1). This experimental design was applied independently for the populations derived from *MBS *and for the populations derived from *F*252. Each family was represented by 100 seeds produced by the selfing of each individual selected at the previous generation. Family' seeds were distributed into 4 blocks (25 seeds of each family per block). Each block was divided into two plots of 11 rows, a Late plot with the 10 Late families and one control, and an Early plot with the 10 Early families and one control. The control consisted in plants from the initial seed lot. Families and control were randomized within the plots. There were 25 plants per row, 25000 plants/ha and 80 cm between the rows. In order to control for systematic environmental effects in all directions, Early and Late plots alternate along the blocks. Within each row, the 3 earliest plants (except the border ones) were selfed in the Early populations, and the 4 to 5 latest plants (except the border ones) were selfed in the Late populations. Kernels were harvested and weighted, and seeds were stored in a cool chamber.

Selection: Since 1997, in each population, the 10 most extreme individuals for selfing date with the highest kernel weight were selected with some additional constraints: (i) we did not select more than two plants of the same row; (ii) we did not select more than three plants of the same family; and (iii) we maintained at least two lineages among the 3 deriving from individuals selected in 1993. Genealogies of the selected individuals are shown in Figure 2.

### Phenotypic evaluation trials

All individuals selected during the first 7 generations of the experiment were evaluated in a 2 years field trial (in 2004 and 2005) at Gif sur Yvette (France). All seeds were produced in the nursery (in 2003) from 25 *S*_1 _progenies for each selected individual of the genealogy. *S*_2 _seeds obtained by selfing each *S*_1 _individual were harvested in bulk for each genotype and constituted the seeds lots used in the evaluation trials. In addition, we used two different seed lots to represent the initial inbreds *F*252 and *MBS*: *S*_2 _seeds from the initial seed lots produced in the nursery in 2003, and commercial seeds. Both were used as control in the evaluation trial. All genotypes were sown in a 4 randomized block design. There were 2 blocks for the genotypes derived from *F*252, and 2 blocks for the genotypes derived from *MBS*. Data from *F*252 and *MBS *were analysed separately. Blocks were further subdivided into 24 sub-blocks. Each sub-block contained 6 plots encompassing 80 plants of the same genotype distributed in 2 rows. Female flowering time was recorded as the date (in days after July 1st) at which 50% of plants within a plot were silking. In total, we evaluated 114 and 115 genotypes derived from *F*252 and *MBS *respectively. The initial inbred lines *F*252 and *MBS *were used as control plots. Altogether, there were at least two control plots in each sub-block of the experimental design. Phenotypic data obtained from the evaluation trials were analysed with R package [66].

Control plots were used to better control for environmental fluctuations and to estimate the effects of the year of experimentation (*y*) and the effects of the sub-block (*c*(*y*)). Phenotypic values of  the controls were decomposed as follow:

where *μ*^*control *^is the average value of the controls,  is the effect of the year of experimentation estimated from the control plots (*l *= 2004, 2005),  is the sub-block effect (*p *= 124) at year *l*,  is the seed lot (*s *= '*Initial*', '*Commercial*'), and  is the residual, with *n *indexing the replicates sharing the same sub-block. Subsequently, phenotypic data (*Y*_*lpn*_) measured on the genotypes of the selection experiment were corrected as follow:

and all the analyses were performed on corrected data *Z*. The analysis of phenotypic variation within the genealogies was performed using the following model:(1)

were *μ *is the average; *y*_*l *_is the effect of the year of experimentation (*l *= 2004, 2005); *b*(*y*)_*lm *_is the block effect (*m *= 1, 2) at year *l *of experimentation; *pop*_*i *_is the effect of the population (*i *= *Late, Early*); *gen*_*j *_is the effect of the generation of selection (*j *= *G*1.. *G*7); (*pop *: *gen*)_*ij *_stands for the interaction between population *i *and generation of selection *j*; and *G*(*pop *: *gen*)_*ijk *_is the genotypic value of the individual *k *of population *i *at generation *j*. Within each population and generation of selection, genotypic values were considered as random variables. Contrasts were used to estimate population means at each generation as  and genotypic values as . After correcting for the year of experimentation and block effects, a separate analysis was conducted on each population at each generation to estimate the genetic variance , the residual variance  and the within population broad-sense heritability for flowering time:(2)

A last separate analysis was performed in each population to estimate the average response to selection *R*_*i *_of population *i *as the slope of the regression of *Z *values over the generations of selection *g*_*j*_, varying from 1 to 7:(3)

### Estimating mutational heritability

We modelled the response to selection and the underlying raise of genetic variation observed in each population, resulting from new mutations, using a similar approach as [33] and [67] and supposing an infinitesimal model. The expected response to selection at generation *g *is given by the Breeder's equation [61]:

where  is the intensity of selection,  is the additive genetic variance, and *σ*_*p *_the phenotypic standard deviation.

Because we performed selfing on an initially inbred material throughout the selection experiment, we considered a haploid population to model the effect of random genetic drift. We supposed that, at each generation, while the genetic variance is depleted by random genetic drift, additional genetic variance is generated by new mutations. We neglected the depletion of genetic variance caused by selection. We also supposed that all the genetic variance is additive, so that:(4)

where  is the effective size of the population at generation *g*, and  is the input of new variation by mutation at each generation.

Iterating equation (4) until generation *g *- 2 gives

while iterating (4) until generation 0 gives(5)

Let , with *k*_0 _= 1, and , being two terms accounting for random genetic drift, the above recursion reduces to:(6)

Here,  > 0 represent the initial genetic variation coming from residual polymorphism within the initial inbred lines.

Phenotypic variation was modelled as the sum of additive genetic effects and a residual term due to environment (*P *= *A *+ ϵ), so that . We defined the mutational heritability as the ratio of the genetic variance created by new mutations at each generation over the residual variance: . We also defined the initial heritability , which accounts for standing genetic variation, as the ratio of the initial genetic variance over the residual variance: . In the following,  is expressed as a fraction of either  or . The recursion for the phenotypic variance was then obtained using (6):(7)

The average response to selection per generation was estimated as , where *G *is the number of generations. According to (6) and (7), the expected response to selection is(8)

After some rearrangement, (8) gives:(9)

(9) was used to estimate the mutational heritability from the observed response to selection in each population. The parameters of (9) were determined as follows: *G *is the number of generations of selection. Because we have no information about the effective population size in the initial seed lot, and because of the small number of individuals selected in 1993, we considered the year 1993 as generation 0, and *G *= 7 as the total number of generations. *R *was considered as a random variable following a Gaussian , where  is the observed response estimated as described in **phenotypic evaluation trials**, and *s.e*.() is the corresponding standard error.  is the selection intensity and depends on the proportion *p*% of selected individuals at each generation, *i.e *it is the mean of the *p*% best values drawn from a Gaussian (0, 1) distribution as given in [68]. At each generation, 100 selfed progenies of each of the 10 selected individuals were sown and observed. The corresponding selection intensity is  = 2.67.  is the variance between individuals having the same genotype. It was considered as a random variable following a uniform distribution ranging from 0.6 days to 12 days. This interval of variation was chosen from values estimated during the selection experiment between 1997 and 2002. *k*_(*g*) _and Π_(*g*) _are related to the effective population size and are computed as described above. Because the pedigrees of the selected individuals are known, we use a similar reasoning as in [4] to estimate the effective population size of each population at each generation from the real population size (*N*), and the variance (*V*(*o*)) of the offspring number:(10)

 depends on the standing genetic variance  at the beginning of the selection. Because the response to selection depends on the total genetic variance, the higher , the lower the estimates of mutational heritability. As the initial genetic variance is unknown, we computed different estimations of the mutational heritability () and the initial heritability ().

The first estimation (Model 1) posits that , and that all the genetic variation comes from mutations that appeared after the beginning of the selection experiment.  under Model 1 was estimated from (9). The second estimation (Model 2) instead posits no mutational variance ( = 0) so that all the genetic variation comes from standing variation measured by ().  under Model 2 was estimated from (8) as follows:(11)

A last estimation (Model 3) combined the two sources of variation (both standing and mutational) and was computed from (9) by choosing the highest value of  which yields a non-zero lower bound for the 5% confidence interval for .

Under Model 1 and Model 3, we used an *EM *iterative algorithm to estimate the mutational heritability from (9). At each iteration, the E-step consists in equating  to its previous value , and the M-step consists in computing the new value of  with equation (9). The initial value was set to , and the *EM *algorithm was reiterated until convergence. The same procedure was applied to estimate  under Model 2 using (11). In order to take into account some uncertainty about the response to selection and the residual variance, the estimation procedure was repeated 10,000 times for each population, with a different value of *R *and *σ*_*e*_. The mutational heritability was estimated as the average value over the 10,000 simulations, and the distribution was used to construct a 5% confidence interval.

### Monte-Carlo simulations and Model testing

Genetic models 1, 2 and 3 described above differ for underlying assumptions about the sources of variation generating the observed response to selection. These assumptions affect primarily the dynamics of the response to selection. For example, if the only source of genetic variation comes from residual polymorphism (Model 2), we expect it to be rapidly exhausted by the combined effects of drift and selection. In contrast, continuous input of genetic variation by mutation (Model 1 and 3) is expected to make the response more linear through time. To test if our experimental results could be explained solely by the segregation of initial polymorphisms without any mutation, Monte-Carlo simulations of the selection experiment were performed using the heritability () estimated under Model 2.

To capture uncertainty in the amount of initial genetic variation, each simulation started by drawing a pair of values (, ) in the empirical distribution produced by Model 2 from experimental data (see above). Because individuals from the last generation all derived from two lineages originating at *G*1 (Figure 2) in three out of the four populations, we started the simulations at generation *G*_1 _with two individuals displaying *n*_*P *_diallelic polymorphic loci randomly distributed on ten chromosomes of 150 *cM*, (*n*_*P *_- *n*_*H*_) of them being homozygous within the individuals but differing between the individuals, the *n*_*H *_remaining ones being heterozygous in both lines. At each locus *l*, allelic effects *a*_*l *_were drawn randomly in an exponential distribution of rate one, and the two alleles only differed by the direction of their effect on the trait. Letting *p*_*l *_be the frequency of the positive allele at locus *l*, the additive genetics variance equals to

Allelic effects were rescaled so that the initial genetic variance is equal to  (scaling coefficient ). In order to mimic the field experiment, each individual produced 100 offsprings.

Reproduction by selfing was simulated by randomly drawing 100 couples of gametes in each individual of the population (leading to 200 selfing seeds in *G*_2 _and 1000 in the next generations *G*_3 _to *G*_7_).

Recombination was produced by drawing crossing-over positions on each chromosome in an exponential distribution of rate 1, *i.e*. Poisson distribution of crossing-overs [69]. In most cases we supposed additivity between and within loci. Hence the phenotypic value of one individual was estimated as the sum of its allelic effects plus a residual term drawn in a Gaussian distribution with variance . The next generation was produced by selecting the best 10 individuals on the basis of their phenotypic values. The average phenotypic value *Z*_*jk *_at generation *G*_*j *_of each selected individual *k *was measured from its 100 offsprings.

These values, together with different summary statistics indicating changes through time of the level of genetic polymorphism were stored at the end of each simulation. For each set of parameters (*n*_*P*_, *n*_*H*_, ), 500 simulations were performed. For each simulation *i*, average phenotypic values were used to estimate the response to selection *r*_*i *_as in (3). Conditionally to the parameters (*n*_*P*_, *n*_*H*_, ) and the uncertainty on , the distribution of *r*_*i*_'s over the 500 runs gives the possible outcomes of the selection experiment without *de novo *mutations. We expect the simulated distribution to mimic the observed response to selection, so that the experimental response *R *(Table 2) that was used to generate the simulations should be included in the simulated distribution. If this is not the case, this means that at least one of the hypotheses is false, *i.e*. either the actual number of initial polymorphisms is different from the simulated one, or gene action is not additive, or new mutations occurred in the actual experiment. The *P value *of the observed response is computed as

In addition, given both the importance of genetic drift and selection in our experiments, the initial standing genetic variation is likely to be greatly reduced within a few generations if no mutation occur (Model 2). Therefore, in the absence of new mutations, we expect a change in the rate of response to selection. To seek for such a pattern, we applied a segmented regression model on simulated data *Z*_*jk *_(1 ≤ *k *≤ 10), with one breakpoint occurring at generation *G*_*b *_(2 ≤ *b *≤ 7).

We varied the generation at which the breakpoint occurs between *G*_2 _and *G*_7 _and used the AICc (Akaike's Information Criteria corrected for small sample sizes [46]) to compare the models. Note that the number of parameters used to build the model changes with the position of the breakpoint. If the breakpoint occurs at *G*_2 _or *G*_6_, one of the two segments is reduced to a point and the total number of parameters in the model is four. If instead the breakpoint occurs at *G*_7 _or after, there is a single segment and three parameters. If the breakpoint occurs between *G*_2 _and *G*_7_, there are two segments and the number of parameters is five. Hence the less penalized model is the one with the breakpoint at *G*_7 _or after (leading to a linear response between *G*_1 _and *G*_7_). This procedure of segmented regression was applied to each simulation and the breakpoint that had the minimum AICc value was retained. Over the 500 simulations we computed the numbers of simulations (*n*_2_, *n*_3_, *n*_7_) for which the change in the rate of response to selection occurred at *G*_2_, *G*_3_, ..., *G*_7_. We also applied the segmented regression to the observed data in the four experimental populations (Late-NVL *F*252, Early *F*252, Late *MBS*, Early *MBS*). For each experimental population, we computed AICc weights

These weights give the probability that the change in the rate of the response to selection occurred at generation *G*_*i *_(2 ≤ *i *≤ 7). These probabilities were compared with the corresponding numbers (*n*_2_, ..., *n*_7_) obtained by simulations under the second genetic model.

### RFLP genotyping

We sowed 4 *S*_1 _offspring derived from each of the 40 individuals (10 individuals from each of the Early and the Late populations derived from *F*252 and *MBS*) selected in 2002 (G = 7). In theory, 4 plants were enough to determine without ambiguity the genotype of the parent (the probability of observing 4 homozygous plants for one allele while the parent is heterozygous is 0.25^4 ^= 0.0039). Hundred and sixty plants were grown during the summer 2004 in field at Gif sur Yvette (France). DNA was extracted from frozen mature leaves according to the procedure described in [70] and quantified on 1% agarose gels. Two micrograms of genomic DNA from each sample were digested 3 hours at 37°C using *EcoR*1 (Fermentas) or *Mbo*1 (Fermentas) according to the manufacturer's instructions. Digested fragments were run on 0.8% agarose gels in 1× *TPE *for 16 hours at 30 V. Depurination, denaturation and capillary transfer to charged Nylon membranes, *HybondN *+(Amersham (Arlington Heights, IL)), were carried out according to the protocol described in [49].

Eight cDNA probes previously mapped [49] and homologous to candidate genes for flowering time in maize [45] were used (Table 1). Probes were obtained from direct PCR amplification using universal M13 primers and one unit of QIAGEN Taq polymerase on overnight grown colonies from glycerol stocks. Two additional probes from genes *zf l*1 and *zf l*2 were used for RFLP assay. Both genes have shown to co-localize with QTLs involved in flowering time [45]. Specific primers for each probe were designed using primer 3 from the published genomic sequence [71]. Forward and reverse primers used to amplify *zf l*1 and *zf l*2 probes respectively are (5'-3'): GCCTCTGCGAGCAATGTGAT, TGCTGCTTCCTTCCTCCTAG, CCCATGCTTCAGTCATGTTG, CAGGTCATCTACGTGCGTGT. PCR reactions were performed in 25 *μL *volumes containing 15 - 30 ng DNA template, 1× PCR buffer, 0.2 mM dNTPs, 2 mM MgCl2, 1 unit of QIAGEN Taq polymerase, 0.2 *μM *of each primer. Initial denaturation of DNA template at 95°C for 5 min was followed by 35 cycles of 95°C for 45 sec, 60°C or 56.4°C for 30 sec respectively for *zf l*1 and *zf l*2, and 72°C for 2 min, and a final extension of 72°C for 7 min. PCR products from the 10 probes were purified with Qiaquick Kit (Qiagen) and checked on 1% agarose gels. 40 ng of each probe was 32P-radio labelled by random priming using the Amersham Megaprime DNA labelling system. RFLP hybridization procedure was performed as described in [49].

### Association mapping using the genealogy

As a polymorphism at locus QCK5e06 was found in the Late *F*252 population, we developed an association test using the genealogy of the individuals. This test measures the association between the genotypes at the candidate locus QCK5e06 and the genotypic values for flowering time estimated for each individual. Statistical analyses were carried out using the R package [66].

The values of the additive (*a*) and dominance (*d*) effects associated with the candidate locus were estimated by linear regression:(12)

Where *G*_*jk *_is the average trait value of genotype *k *belonging generation *j*; *μ*_*j *_is the average flowering time calculated across all individuals at generation *j*; *x*_*k *_and *y*_*k *_are indicator variables of the genotype of the individual at the candidate locus; *x*_*k *_= 1 or - 1, and *y*_*k *_= 0 for an homozygous individual, and *x*_*k *_= 0 and *y*_*k *_= 1 for a heterozygous individual; ϵ_*k *_is the residual. In order to get enough power to analyse our data, instead of discarding missing data, we decided to treat them as follows: missing data for the genotype at the candidate locus were treated by triplicating the individuals and weighting each three possible values for (*x*_*k*_, *y*_*k*_) = (1, 0), (0, 1), (- 1, 0) by their probabilities of occurrence knowing the genotype of the ancestor; missing phenotypic values were replaced by the average trait values of the genotypes at the same generation with the same most recent common ancestor (Figure 3). The genotype at the QCK5e06 locus and the genotypic values of each individual of the genealogy were used to estimate the observed values of *a *and *d*, respectively *a*_*obs *_and *d*_*obs*_.

To test the association between flowering time variation and the segregation of alleles at the candidate locus, we asked whether the *a*_*obs *_and *d*_*obs *_could result from random genetic drift along the genealogy, causing a spurious association. Because the individuals are connected through their pedigree, we chose to simulate the null distribution *H*_0 _of random segregation of alleles in the genealogy. Practically, to match with the observed genotypes, we started the simulations with 2 heterozygous individuals at *G*1 at the candidate locus. Each simulation consisted in gene-dropping the two alleles throughout the genealogy of the 59 individuals descending from these two heterozygote parents at *G*1, as shown in Figure 3. At each generation, *g*, the genotype of each individual was drawn at random knowing the genotype of its parent and assuming Mendelian inheritance. Reversion events were neglected. In a second step, we performed association mapping as described above, using the simulated genotypes and the genotypic values of the 61 individuals to estimate *a *and *d *and construct the null distribution. At the end, *a*_*obs *_and *d*_*obs *_were compared to the resulting *H*_0 _distributions.

## Competing interests

The authors declare that they have no competing interests.

## Authors' contributions

CD and AC conceived and designed the study. CD conducted the selection experiment and collected field data. DC, PJ and SJ were in charge of field experiments and seed management. CD and ED performed the quantitative genetics analyses and modelling. MIT conducted the lab work with the help of CR and performed the association analysis with CD. CD, ED, AR and MIT discussed statistical analyses and data interpretation. CD and MIT wrote the manuscript with the help of ED and incorporated critical comments of AC and AR. All authors read and approved the final manuscript.
